# A Multiscale Study of CFRP Based on Asymptotic Homogenization with Application to Mechanical Analysis of Composite Pressure Vessels

**DOI:** 10.3390/polym14142817

**Published:** 2022-07-11

**Authors:** Nan Zhang, Shuai Gao, Meili Song, Yang Chen, Xiaodong Zhao, Jianguo Liang, Jun Feng

**Affiliations:** 1 School of Mechanical Engineering, Nanjing University of Science and Technology, Nanjing 210094, China; znnj9710@163.com (N.Z.); gaoshuai0816@163.com (S.G.); Meili_Song@163.com (M.S.); cyymseven@163.com (Y.C.); 2 College of Mechanical and Vehicle Engineering, Taiyuan University of Technology, Taiyuan 030024, China; zhaoxiaodong0028@link.tyut.edu.cn; 3 National Key Laboratory of Transit Physics, Nanjing University of Science and Technology, Nanjing 210094, China

**Keywords:** CFRP, asymptotic homogenization, multiscale analysis, composite pressure vessel, burst pressure

## Abstract

The application of composites is increasingly extensive due to their advanced properties while the analysis still remains complex on different scales. In this article, carbon fiber reinforced polymer (CFRP) is modeled via asymptotic homogenization employing a representative volume element (RVE) with periodic boundary conditions. A multiscale mechanical model of CFRP is established to bridge the microscopic model, mesoscopic model, and macroscopic model. According to asymptotic homogenization, the coefficients of the material constitutive equation are calculated with volume-averaged stress and strain. Using the homogenized materials properties of CFRP, the tensile experiments of composite layers with the layout of [${\left(0^{\circ }/60^{\circ }/0^{\circ }/-60^{\circ }\right)}_{4}$] are carried out to validate asymptotic homogenization method. The results indicated that the asymptotic homogenization approach can be used to calculate the homogenized elastic moduli and Poisson’s ratio of the whole structure, where the numerical results are basically consistent with test data. The sequent homogenized CFRP laminate model is applied to the mechanical analysis of type III composite pressure vessels, whereby burst pressure is accurately predicted. This work might shed some light on multiscale analysis of composite pressure vessels.

## 1. Introduction

Characterized by lightweight as well as excellent mechanical properties compared to traditional metals, carbon fiber reinforced polymer (CFRP), composed of reinforcements and polymer matrix, has become the most important material in aerospace structures [1,2]. Recently, the commercial markets of CFRP are no longer confined to the aerospace industry but wider industrial sectors e.g., architecture and automobiles [3,4,5]. Such extensive applications have been promoted by the in-depth understanding of the governing physical and mechanical behaviors at various scales [6,7,8].

The homogenization method based on two-scale asymptotic expansion first proposed by Bensoussan et al. has been widely used in predicting the effective properties of composites [9]. Bakhvalov and Panasenko [10] studied the solutions of differential equations with fast oscillating coefficients using standard asymptotic methods to study various processes in media with periodic structures, and the concept of asymptotic expansion is also introduced in detail. Tang et al. [11] proposed a multi-scale modeling framework based on the crystal plastic finite element method, and proved the accuracy of the framework through experiments and computational studies. For three-dimensional braided composites, the establishment of representative volume element (RVE) under periodic boundary conditions based on the homogenization principle and the finite element method can predict the mechanical properties [12,13,14,15,16,17,18,19] and damage mechanism [20,21,22,23,24] of the composites. Meanwhile, the influence of reinforcement content and layering mode on the mechanical properties of the composites can also be studied. For complex composite models, computational costs are saved by combining macroscale and microscale. Tang et al. [25] only carried out microscopic modeling of the region of interest, while the homogenized properties contribute to the other regions to study rack propagation of chopped carbon fiber chip reinforced sheet molding compound composites under fatigue conditions.

The presently emerging generation of hydrogen fuel vehicles uses composite pressure vessels to store hydrogen gas at high pressure to guarantee desired energy density [26]. The filament winding technology has been applied to design high-capacity pressure vessel structures with fibrous composites [16,27]. Composed of an inner aluminum liner winded with outer CFRP bundle layers, type III composite vessels have successfully been utilized for onboard applications of buses and private cars [28]. The composite vessel manufacturing is realized by deploying the CFRP filament in different orientations to stack together reaching high stiffness and strength. In addition, such CFRP structures support around 99% of the internal pressure load. In complex environments, the mechanical properties of CFRP will change, such as fatigue performance [29] and compositional change [30], which is also a challenge to the performance of composite pressure vessels. Hence, a robust and accurate modeling method is in need for composite vessel evaluation and analysis.

Since the diameter of a T700 fiber is only 7 μm, an accurate finite element model for the microstructure of CFRP is impossible [31]. The multiscale model bridging micro to macro composite layers would be a good solution. In numerical studies, the filament winding CFRP layers can be considered as the laminate composite structure. Liu and Zheng [32,33] studied the hydrogen pressure vessel with effective parametric studies to predict burst pressure. Linking micromechanics and continuum mechanics, Nguyen and Simmons [27] build the lamina behavior to analyze mechanical responses in complex filament-wound composite vessel structures, which demonstrated lamina thickness and helical angle effects on burst pressure.

In general, the conventional finite element method directly models the microscale structure of the CFRP which is too complex to handle. The detailed microstructure geometry modeling requires refined meshing elements resulting in tremendous computational cost [34]. The microscale modeling result data can be redundant as failure usually occurs at some specific positions of the CFRP structure. Therefore, multiscale modeling such as asymptotic homogenization (AH) and the representative volume element (RVE) has been developed to bridge the composite mechanical responses in various scales [35]. Predicting effective material properties from microscale upward to mesoscale and macroscale, asymptotic homogenization has been widely applied to investigate CFRP composite materials and structures. In the AH method, the characteristic displacement tensor needs to be computed to evaluate composite heterogeneity. Yuan and Fish [36] developed an ingenious implementation with thermal expansion strain to achieve elastic moduli of composite.

Although AH has gained success in multiscale modeling of composite structures, the filament winding composite vessel analysis can be further improved via two-stage homogenization. The first stage of homogenization is applied to determine CFRP bundle elastic moduli with uniaxially aligned fibers while the filament winding laminates can be homogenized with composite bundle layers in the second stage. In this paper, the asymptotic homogenization method is used for multiscale analysis of CFRP composite, whereby the homogenized stiff matrix of CFRP is calculated via the ABAQUS solver. A uniaxial tensile test of CFRP laminate is carried out to validate the asymptotic homogenization. The mechanical analysis of type III hydrogen storage pressure vessels is performed with homogenized CFRP filament winding layers. Mechanical responses including burst pressure are numerically studied and compared with reported results.

## 2. Asymptotic Homogenization of CFRP

### 2.1. Asymptotic Expansion

**Figure 1:**
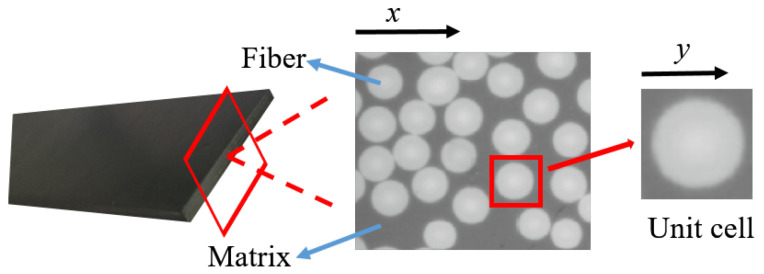
Selection of RVE.

Proposed by Babuska [37] in 1976, the homogenization approach is deemed an effective method to study the macroscopic behavior of a medium through its microscopic properties. By representing the physics and mechanics of the overall composite material structure, the homogenization approach provides a tool to conduct numerical processing of microstructure problems. Consider a piece of periodic heterogeneous macrostructure Ω, the coordinates of the arbitrary point at the macro level are denoted $x=(x_{1},x_{2})$ and in the micro level are denoted $y=(y_{1},y_{2})$ for a 2-D medium in the Cartesian coordinate system, as shown in Figure 1. Connect the macrocoordinate *x* with the micro coordinate *y* by a parameter ϵ:

(1) $$\epsilon =\frac{x}{y}$$

The displacement field *u* by using small parameter ϵ can be expanded as

(2) $$u^{\epsilon }\left(x\right)=u\left(x,y\right)=u^{0}\left(x,y\right)+\epsilon u^{1}\left(x,y\right)+\epsilon^{2}u^{2}\left(x,y\right)+\cdots$$

Using the derivative rule, strain $\varepsilon_{ij}$ corresponding to displacement *u* in Equation (2) is

(3) $$\begin{array}{c} \varepsilon_{ij}\left(u^{\epsilon }\right)=\frac{1}{2}\left(\frac{\partial u_{i}^{\epsilon }}{\partial x_{j}^{\epsilon }}+\frac{\partial u_{j}^{\epsilon }}{\partial x_{i}^{\epsilon }}\right)=\frac{1}{\epsilon }\varepsilon_{ij}^{\left(-1\right)}\left(x,y\right)+\varepsilon_{ij}^{\left(0\right)}\left(x,y\right)+\epsilon \varepsilon_{ij}^{\left(1\right)}\left(x,y\right)+\epsilon^{2}\cdots \end{array}$$

It is assumed that the stiffness coefficient tensor of the material is $C_{ijkl}^{\epsilon }$. In the constitutive relation of the structure, the relationship between stress and strain is expressed as

(4) $$\sigma_{ij}^{\left(n\right)}\left(x,y\right)=C_{ijkl}^{\epsilon }\varepsilon_{ij}^{\left(n\right)}\left(x,y\right)\;\left(n=-1,0,1\right)$$

(5) $$\sigma_{ij}^{\epsilon }=C_{ijkl}^{\epsilon }\varepsilon_{kl}=\frac{1}{\epsilon }\sigma_{ij}^{\left(-1\right)}\left(x,y\right)+\sigma_{ij}^{\left(0\right)}\left(x,y\right)+\epsilon \sigma_{ij}^{\left(1\right)}\left(x,y\right)+\cdots$$

According to Equations (3) and (4), $\sigma_{ij}^{\left(-1\right)}$ and $\sigma_{ij}^{\left(0\right)}$ can be obtained.

(6) $$\sigma_{ij}^{\left(-1\right)}\left(x,y\right)=C_{ijkl}^{\epsilon }\frac{\partial u_{k}^{\left(0\right)}}{\partial y_{l}}$$

(7) $$\sigma_{ij}^{\left(0\right)}\left(x,y\right)=C_{ijkl}^{\epsilon }\left(\frac{\partial u_{k}^{\left(0\right)}}{\partial x_{l}}+\frac{\partial u_{k}^{\left(1\right)}}{\partial y_{l}}\right)$$

Considering the basic equation of linear elasticity problem, the equilibrium equation is introduced:

(8) $$\sigma_{ij,j}+f_{i}=0\;in\;\Omega$$

By combining the equilibrium equation, Equation (5) can be converted to

(9) $$\begin{array}{c} \epsilon^{-2}\frac{\partial \sigma_{ij}^{\left(-1\right)}}{\partial y_{j}}+\epsilon^{-1}\left(\frac{\partial \sigma_{ij}^{\left(-1\right)}}{\partial x_{j}}+\frac{\partial \sigma_{ij}^{\left(0\right)}}{\partial y_{j}}\right)+\epsilon^{0}\left(\frac{\partial \sigma_{ij}^{\left(0\right)}}{\partial x_{j}}+\frac{\partial \sigma_{ij}^{\left(1\right)}}{\partial y_{j}}+f_{i}\right)+\epsilon^{1}\left(\frac{\partial \sigma_{ij}^{\left(1\right)}}{\partial x_{j}}+\frac{\partial \sigma_{ij}^{\left(2\right)}}{\partial y_{j}}\right)+\epsilon^{2}\cdots =0 \end{array}$$

For ϵ→0, a series of perturbation equations are as follows:

(10) $$\frac{\partial \sigma_{ij}^{\left(-1\right)}}{\partial y_{j}}=0$$

(11) $$\frac{\partial \sigma_{ij}^{\left(-1\right)}}{\partial x_{j}}+\frac{\partial \sigma_{ij}^{\left(0\right)}}{\partial y_{j}}=0$$

The limit relation of periodic function ϕ(y) is

(12) $$\lim_{\epsilon \to 0^{+}}\int_{\Omega^{\epsilon }}\phi \left(x/\epsilon \right)dV=\frac{1}{Y}\int_{\Omega }\left(\int_{Y}\phi \left(y\right)dY\right)dV$$

A characteristic function $\left(\chi_{k}^{mn}\left(y_{i}\right)\right)$ is introduced to relate the macro displacement to the first order displacement. The derived characteristic function satisfies

(13) $$\frac{\partial }{\partial y_{j}}\left(C_{ijkl}\frac{\partial \chi_{k}^{mn}}{\partial y_{l}}\right)=-\frac{\partial C_{ijmn}}{\partial y_{j}}$$

Considering the symmetry of kl, it is assumed that mn is constant.

(14) $$\frac{\partial }{\partial y_{j}}\left[C_{ijkl}\frac{1}{2}\left(\frac{\partial \chi_{k}^{mn}}{\partial y_{l}}+\frac{\partial \chi_{l}^{mn}}{\partial y_{k}}\right)\right]=-\frac{\partial C_{ijmn}}{\partial y_{i}}$$

The thermal stress method is used to solve the characteristic function. A change in temperature produces thermal deformation. Equation (14) is converted to

(15) $$\frac{\partial \sigma_{ij}^{\left(mn\right)}}{\partial y_{j}}=0$$

where

(16) $$\sigma_{ij}^{\left(mn\right)}=C_{ijkl}\left[\frac{1}{2}\left(\frac{\partial \chi_{k}^{mn}}{\partial y_{l}}+\frac{\partial \chi_{l}^{mn}}{\partial y_{k}}\right)+I_{klmn}\right]$$

(17) $$I_{klmn}=\left(\delta_{mk}\delta_{nl}+\delta_{nk}\delta_{ml}\right)/2$$

Introduce temperature variable ΔT

(18) $$I_{klmn}=-\kappa_{kl}^{mn}\Delta T$$

where $\kappa_{kl}^{mn}$ is coefficient of thermal expansion, and temperature change value ΔT = 1. The original homogenization equation is transformed into thermal stress equation.

(19) $$\begin{array}{c} C_{ijmn}^{H}=\frac{1}{\left|Y\right|}\int_{Y}\sigma_{ij}^{\left(mn\right)}dY=\frac{1}{\left|Y\right|}\int_{Y}C_{ijkl}\left[\frac{1}{2}\left(\frac{\partial \chi_{k}^{mn}}{\partial y_{l}}+\frac{\partial \chi_{l}^{mn}}{\partial y_{k}}\right)+I_{klmn}\right]dY \end{array}$$

where $\kappa_{kl}^{mn}$ is a fourth-order tensor, kl represents row and mn represents column, and thermal deformation $\varepsilon_{klmn}^{T}=\kappa_{kl}^{mn}\Delta T$. The coefficient of thermal expansion $\kappa_{kl}^{mn}$ can be expressed by the Voigt model as

(20) $$\kappa_{kl}^{mn}=\left[\begin{array}{cccccc} -1 & 0 & 0 & 0 & 0 & 0\\ 0 & -1 & 0 & 0 & 0 & 0\\ 0 & 0 & -1 & 0 & 0 & 0\\ 0 & 0 & 0 & -1 & 0 & 0\\ 0 & 0 & 0 & 0 & -1 & 0\\ 0 & 0 & 0 & 0 & 0 & -1 \end{array}\right]$$

In ABAQUS, it is convenient to model and achieve temperature change. A key aspect of solving a unit cell problem is the implementation of periodic boundary conditions. Face-to-face constraints are considered appropriate, where each node on a slave surface is constrained to have the same motion as the closest point on the master surface. The properties of a composite at the micro level can be solved by the homogenization approach, and simplified modeling of composite material layers of pressure vessels also needs this theory. The homogenization approach can also be used to calculate the homogenized parameters of the laminates with different layering modes.

The asymptotic homogenization implementation is developed for computing the integral form characteristic displacement tenor and the effective linear elasticity. Firstly, a finite element model of RVE is created and meshed, which includes node information and mechanical properties of different constituents. Secondly, the material properties and thermal expansion coefficient are set for different components and apply periodic boundary conditions to the parallel surfaces. Then a unit temperature change is applied to the model. Finally, the derived characteristic displacement tensor is used to compute the effective material properties via the ABAQUS solver with a subroutine.

### 2.2. Multiscale Analysis

#### 2.2.1. Microscopic Model

**Figure 2:**
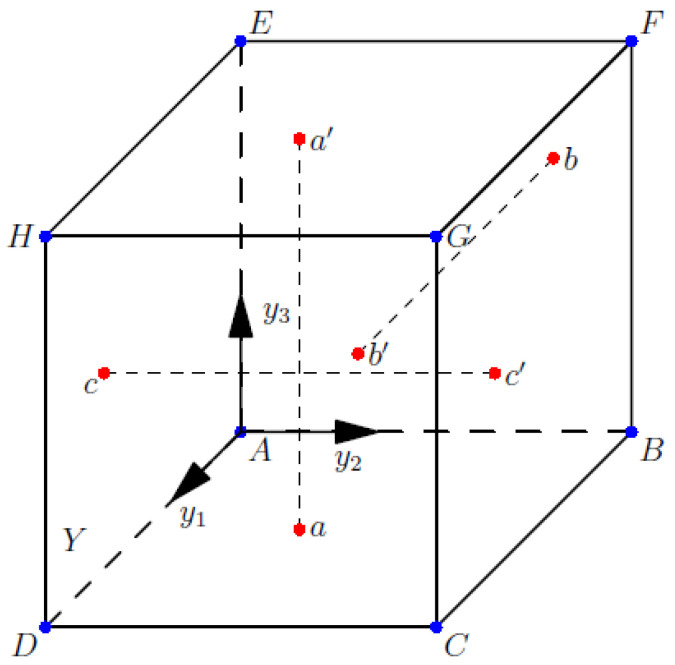
Periodic boundary conditions.

Considering periodic boundary conditions, the global properties are represented by partial properties in the model environment. Assuming that the unit cell is hexahedral in shape, A, B, C, D, E, F, G, and H are the vertices of a hexahedron. The characteristic function at the vertex is $\chi_{i}^{mn}=0$. In Figure 2, a is an arbitrary point in plane ABCD, and a’ is the point corresponding to an in plane EFGH where a and a’ have the same characteristic function, as do b and b’ and c and c’. Based on the homogenization approach, the coefficients of the structure are solved by unit cells, so as to obtain mechanical properties. CFRP is composed of carbon fiber and epoxy. The mechanical properties of carbon fiber and epoxy differ greatly as shown in Table 1.

**Figure 3:**
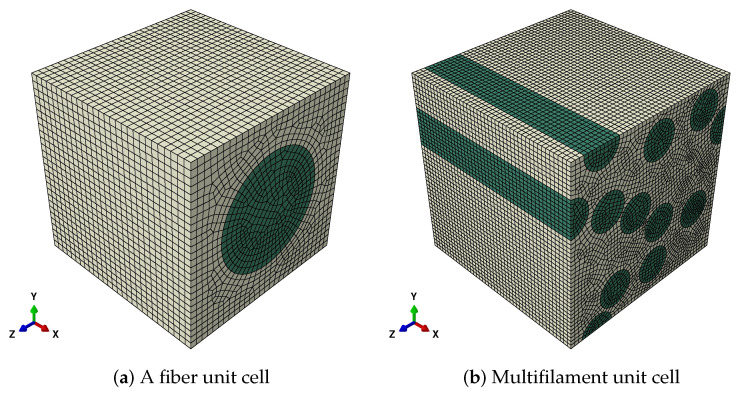
Unit cell.

Different parameters are assigned to different materials in ABAQUS, and the periodic boundary conditions are set for the model [36]. Each node on a master surface constrains the same motion as the closest point on the slave surface. With an initial temperature field of 0, the temperature field is set to 1 in Step 1, and the expansion coefficient in the six loading cases is used. The static universal analysis method is adopted. The unit cell (a fiber) model is shown in Figure 3a where 26460 C3D8R elements are generated.

**Figure 4:**
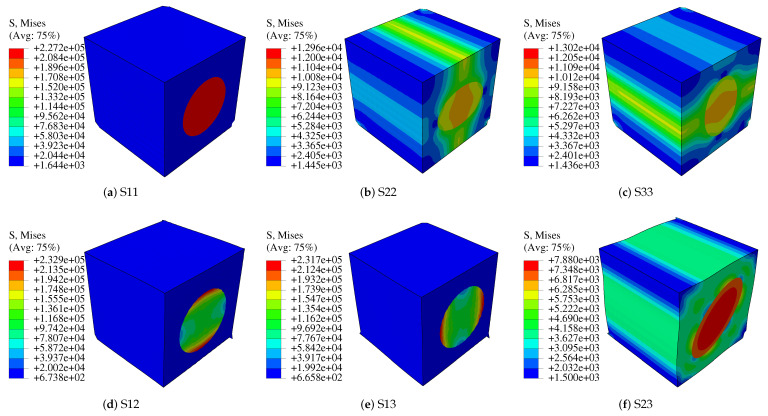
Characteristic stress distribution in each direction.

**Figure 5:**
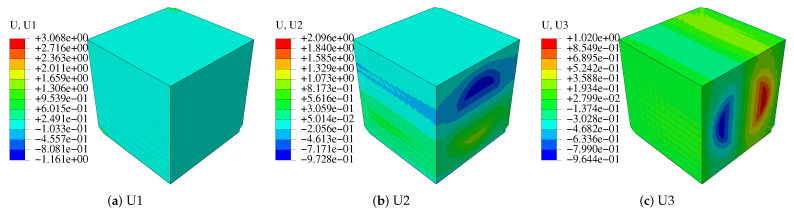
Characteristic displacement χ in the direction of 11.

Using a Python subroutine, the step is carried by submitting a single job. In ABAQUS/CAE, the visualization module can be used to output von-Mises stress and displacement influence functions as shown in Figure 4 and Figure 5. Figure 5 shows the characteristic displacement contour under the loading along the fiber direction of 11. Finally, the homogenized stiffness matrix of the fibrous composite in Equation (21) is calculated.

(21) $$O_{ij}={\left[S_{11}\;S_{22}\;S_{33}\;S_{12}\;S_{13}\;S_{23}\right]}^{-1}$$

CFRP is an anisotropic material, and Equation (22) is the flexibility matrix of constitutive relation, where $E_{1}$, $E_{2}$, and $E_{3}$ are the elastic moduli in directions 1, 2, and 3 respectively. $G_{23}$, $G_{13}$, and $G_{12}$ are the shear moduli of planes 2-3, 3-1, and 1-2 respectively. $\nu_{ij}$ is poisson’s ratio of transverse strain in *j* direction when stress is acting in *i* direction. For orthotropic materials, there are only nine independent constants because the constitutive relation is symmetric. Only $\nu_{12}$, $\nu_{13}$, and $\nu_{23}$ need further study, while $\nu_{21}$, $\nu_{31}$, and $\nu_{32}$ can be expressed by the other three Poisson’s ratios and elastic moduli.

(22) $$D_{\mathrm{ij}}=\left[\begin{array}{cccccc} \frac{1}{E_{1}} & \frac{-\nu_{21}}{E_{2}} & \frac{-\nu_{31}}{E_{3}} & 0 & 0 & 0\\ \frac{-\nu_{12}}{E_{1}} & \frac{1}{E_{2}} & \frac{-\nu_{32}}{E_{3}} & 0 & 0 & 0\\ \frac{-\nu_{13}}{E_{1}} & \frac{-\nu_{23}}{E_{2}} & \frac{1}{E_{3}} & 0 & 0 & 0\\ 0 & 0 & 0 & \frac{1}{G_{23}} & 0 & 0\\ 0 & 0 & 0 & 0 & \frac{1}{G_{13}} & 0\\ 0 & 0 & 0 & 0 & 0 & \frac{1}{G_{12}} \end{array}\right]$$

In ABAQUS, the expansion coefficient in each case is set to −1, and the homogenized stiffness matrix of a fiber unit cell is obtained by output database. The coefficients for homogenized stiffness matrix of a fiber unit cell is shown as

(23) $$\begin{array}{c} C_{\mathrm{single}}=\left[\begin{array}{cccccc} 380800 & 7118 & 7119 & -16 & -17 & -14\\ 7118 & 13003 & 6968 & -14 & -11 & -20\\ 7119 & 6934 & 13007 & -11 & -15 & -19\\ -16 & -14 & -11 & 13583 & 3 & -0.8\\ -17 & -11 & -15 & 3 & 14 & -0.7\\ -14 & -20 & -19 & -0.8 & -0.7 & 27801 \end{array}\right] \end{array}$$

Invert the stiffness matrix, so the flexibility matrix is shown as

(24) $$\begin{array}{cc} \; & S_{\mathrm{single}}=C_{\mathrm{single}}^{-1}=e-3\left[\begin{array}{cccccc} 0.0132 & -0.00474 & 0.0047 & 0 & 0 & 0\\ -0.0047 & 0.1095 & -0.0561 & 0 & 0 & 0\\ -0.0047 & -0.0561 & 0.1095 & 0 & 0 & 0\\ 0 & 0 & 0 & -0.074 & 0 & 0\\ 0 & 0 & 0 & 0 & 0.074 & 0\\ 0 & 0 & 0 & 0 & 0 & 0.36 \end{array}\right] \end{array}$$

**Figure 6:**
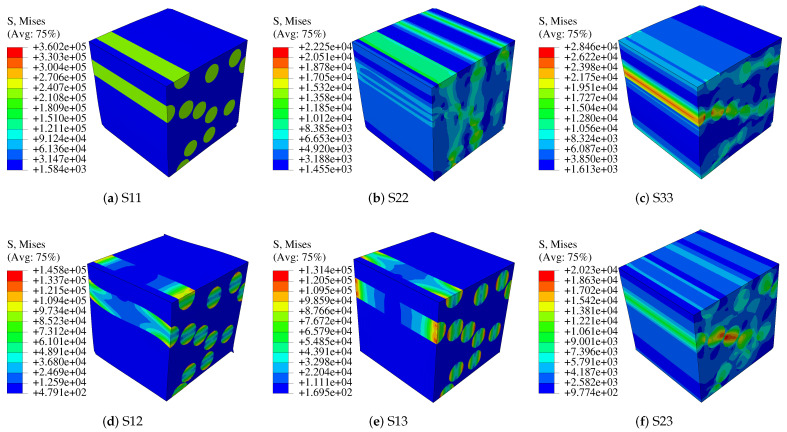
Characteristic stress distribution in each direction (fibers).

According to the corresponding relationship between engineering constants and the values in Equation (22), homogenized mechanical properties of a single fiber unit cell can be obtained. Using the same approach to verify the consistency of single fiber unit cell and fibers unit cell, the fibers unit cell simulation with periodic boundary conditions is established in ABAQUS. The cell is shown in Figure 3b with 109,120 elements. The solution method is the same as that of a single fiber unit cell, and the output von-Mises stress is shown in Figure 6. Fibers unit cell and a fiber unit cell are solved in the same way, and the mechanical property parameters of the two kinds of unit cells are obtained as shown in Table 2.

The calculation results of single fiber unit cell and randomly distributed fibers unit cell are close. Due to the interaction between fibers, the elastic modulus value of randomly distributed fiber and single fiber cells have a certain difference but are also within an acceptable range. Poisson’s ratios are acceptable. We have previously conducted a uniaxial tensile experiment of carbon fiber bundles, and the experiment results of ultimate strength showed a deviation between 5 and 11.8%. Poisson’s ratios are also acceptable. So the feasibility of asymptotic homogenization is verified.

#### 2.2.2. Mesoscopic Model

**Figure 7:**
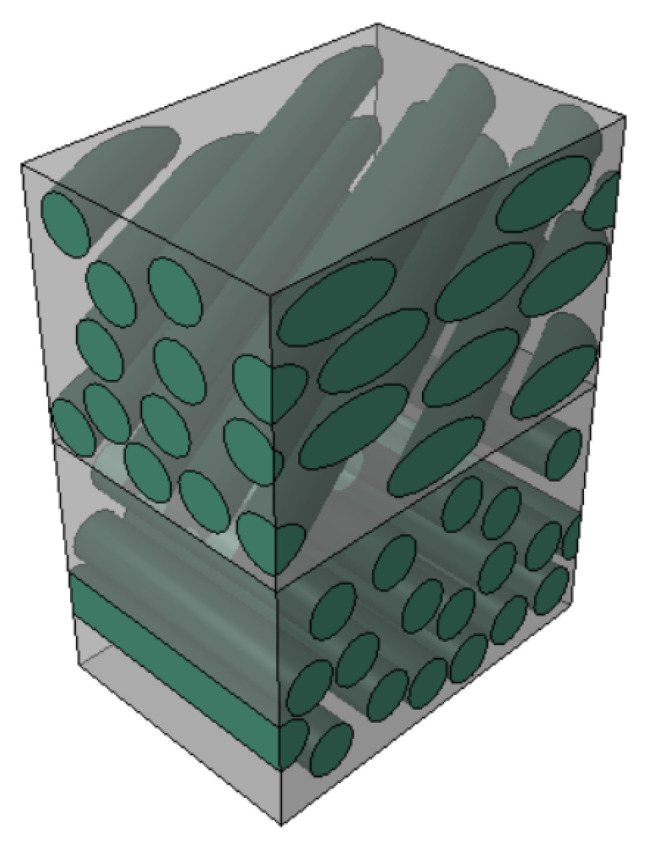
Mesoscopic model.

**Figure 8:**
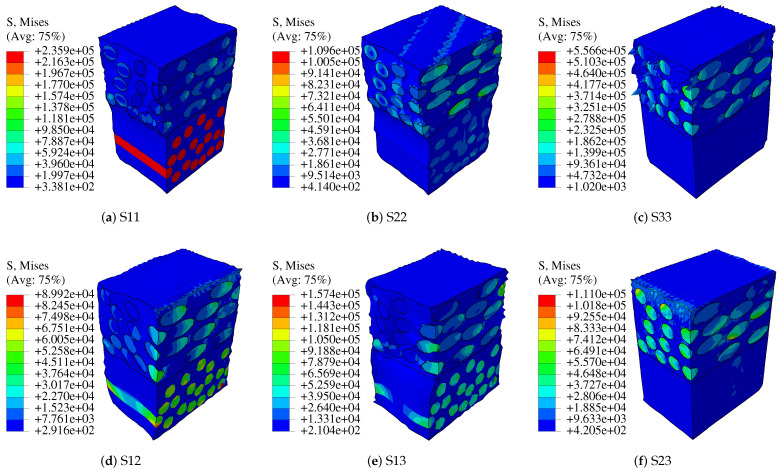
Characteristic stress diagram of mesoscopic model.

In this work, two layers of CFRP of $0^{\circ }$ and $60^{\circ }$ are taken as a whole to study its elastic modulus. With 32% fiber volume fraction, the model is shown in Figure 7 and the material properties of the model are shown in Table 1. Setting boundary conditions, the elastic modulus of the overall model is calculated using the foregoing asymptotic homogenization approach. The von-Mises stress patterns in six cases are shown in Figure 8.

The same method as the microscopic model is used to calculate some material parameters of the mesoscopic model, as shown in Table 3. For general engineering requirements, using different laying ways of CFRP, the modeling process and calculation in engineering are often complicated. Through the homogenization approach, different composite layers can be regarded as a whole, and the material parameters of the whole can be calculated, which can reduce the complexity of the simulation calculation.

#### 2.2.3. Validation on the Macroscopic Scale

**Figure 9:**
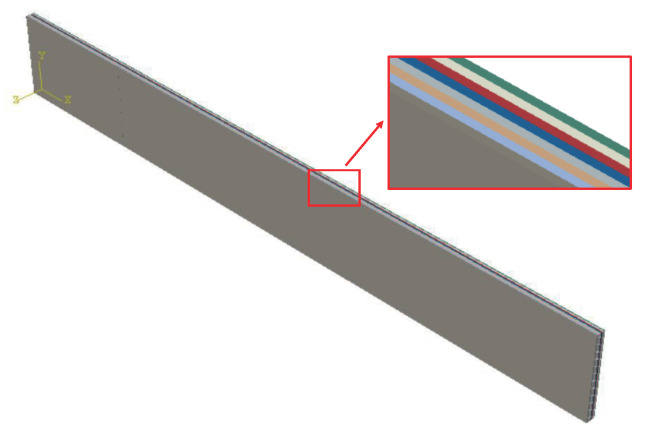
Macroscopical model.

On the macroscopic scale, homogenized material parameters are applied to the model in ABAQUS, and the layers of 0${}^{\circ }$ and 60${}^{\circ }$ are regarded as a whole. In ABAQUS, the Hashin criterion and Puck criterion are used to calculate the maximum failure stress value of the macroscopic model, and the numerical results are compared with the experimental data. The numerical model was built with eight layers in total. Each layer of the model is equivalent to two layers of the test sample, as shown in Figure 9. The material parameters adopted for simulation are shown in Table 3. One end of the model is fixed and the other end is stretched until the model breaks. After the simulation, the ultimate strengths of the fracture models are compared with test results to validate the homogenized material parameters.

## 3. Experimental Program

### 3.1. Specimen Preparation

A tensile test of CFRP laminate is conducted to examine the material properties and validate the homogenization methods. A strip of multidirectional composite laminate [($0^{\circ }$/$60^{\circ }$/$0^{\circ }$/$-60^{\circ }$)${}_{4}$] consisting of 33% carbon fiber and 67% epoxy in terms of volume has a size of 210 mm × 210 mm. The sample of the CFRP plate is Toray T700SC-12K carbon fiber prepreg. After layering and bonding, Teflon film is used to cover both sides to complete the production of preprocessed parts.

The processing equipment of thermosetting processing is 50 tons 400 mm automatic program intelligent vacuum high-temperature hot press, and the model is HBSCR-50T/350AV. The preprocessed sample is placed in a specific metal frame, and the edge of the sample fits with the internal frame of the metal frame. A fully covered metal plate is placed above the sample to fix the preprocessed sample. During the thermosetting process, the vacuum value of the equipment is kept within the range of −0.082 MPa ∼−0.078 MPa. In Step 1, CFRP is heated to 80 °C and maintained for 360 s, at which time the product pressure is 0.1 MPa. In Step 2, CFRP is heated to 120 °C and maintained for 2400 s, at which time the product pressure is increased to 0.35 MPa and maintained until the end of the molding. In Step 3, CFRP is cooled at a slow rate to 60 °C to prevent warping. After forming, the CFRP plate with the size of 210 mm × 210 mm × 2.75 mm is taken out. It is left standing for 24 h for cutting.

**Figure 10:**
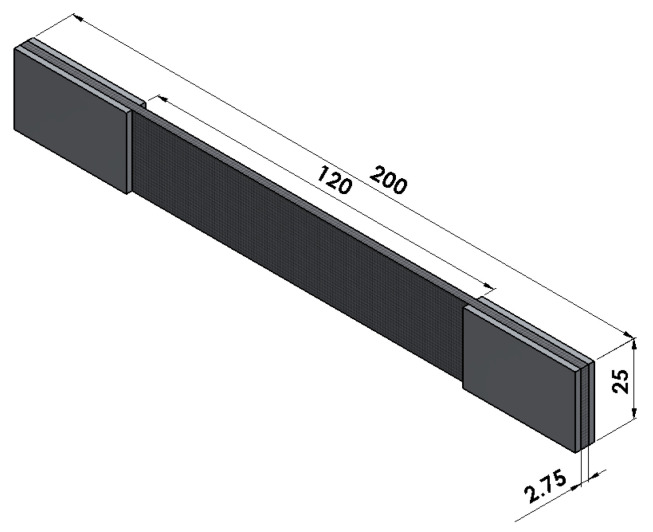
Specimen model (mm).

Using CNC (computer numerical control) to cut CFRP can guarantee high machining quality. Due to the size limitation of the CFRP plate, the tensile sample size after cutting is 200 mm × 25 mm × 2.75 mm, as shown in Figure 10. A 1060 aluminum sheet with a size of 40 mm × 25 mm × 2.75 mm is used as a reinforcement sheet. Both ends of specimens are adhered to stiffeners. Different specimens are used to measure the longitudinal and transverse elastic moduli. The product is cut in different vertical directions with the shape and size of the specimens unchanged. In this way, tensile specimens in both transverse and longitudinal directions can be obtained. The uniaxial tensile test is carried out according to the standard [38].

### 3.2. Tensile Experiment

**Figure 11:**
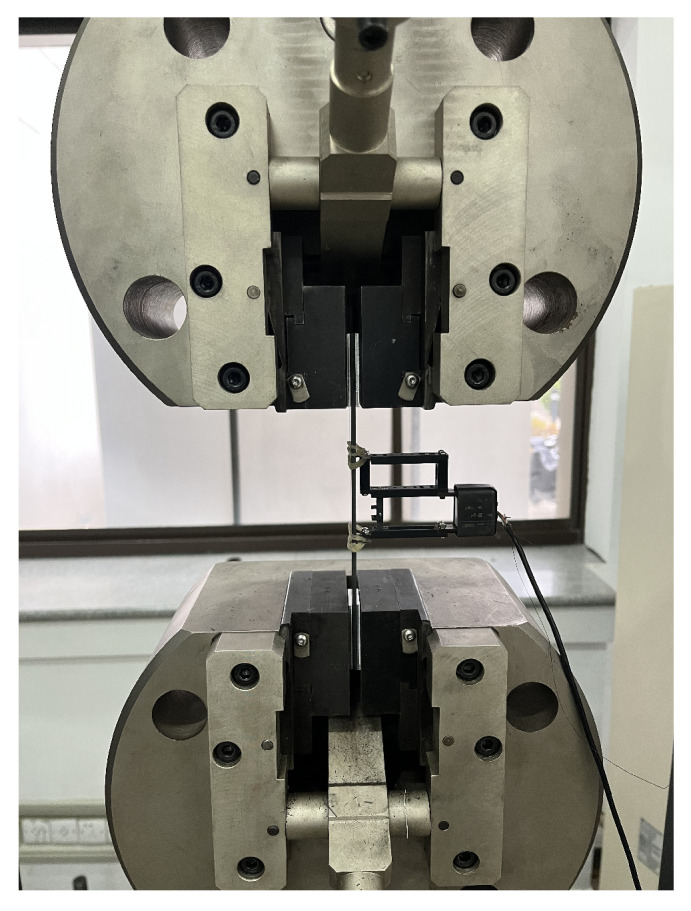
Experimental fixture and displacement meter.

**Figure 12:**
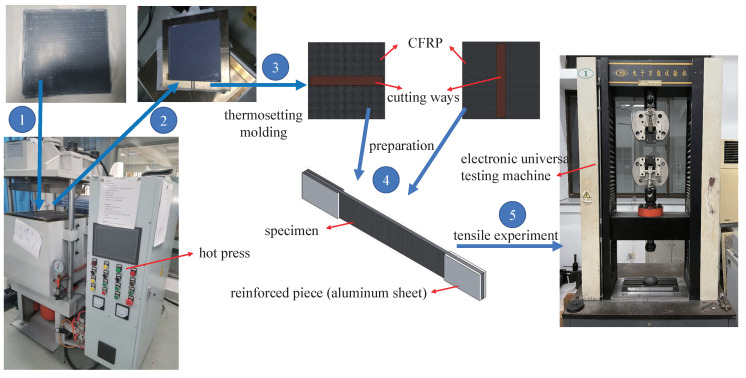
Specimen preparation and test process.

The tensile experiment of CFRP is divided into two parts. The uniaxial tensile experiments are carried out in the directions of [( $0^{\circ }$/$60^{\circ }$/$0^{\circ }$/$-60^{\circ }$)${}_{4}$] and [( $90^{\circ }$/$30^{\circ }$/$90^{\circ }$/$-30^{\circ }$)${}_{4}$] respectively. Three effective test samples are taken from each part to measure the longitudinal and transverse elastic modulus of CFRP samples. In the experiment process, if the sample is debonding or has a root fracture, then it is regarded as an invalid sample. A 200 kN electronic universal testing machine is used for the test equipment. The fixture and displacement meter are shown in Figure 11. The displacement meter is used to measure the elongation of the sample during the test. The displacement-controlled loading method is adopted in the test, and the loading rate was 1 mm/min [38]. Sample preparation and the test process are shown in Figure 12.

### 3.3. CFRP Laminate Homogenization Theoretical Study

#### 3.3.1. Rule of Mixtures

##### Longitudinal Modulus

It is assumed that the strain values of the CFRP, fiber and the matrix are equal,

(25) $$\epsilon_{c}=\epsilon_{f}=\epsilon_{m}$$

and the total force acting on CFRP is equal to the sum of the forces acting on the fiber and matrix,

(26) $$F_{c1}=F_{f}+F_{m}$$

(27) $$F=\frac{\sigma }{A}$$

where ϵ is strain; *F* is force; σ is stress; *A* is cross sectional area.

(28) $$\sigma_{c1}A_{2}=\sigma_{f}A_{f}+\sigma_{m}A_{m}$$

(29) $$\sigma_{c1}=\sigma_{f}\frac{A_{f}}{A_{c}}+\sigma_{m}\frac{A_{m}}{A_{c}}$$

By referring to Hooke’s Law and fiber volume fraction $V_{f}$ and matrix volume fraction $V_{m}$, the following equation can be obtained,

(30) $$E_{c1}=E_{f}V_{f}+E_{m}V_{m}=E_{f}V_{f}+E_{m}\left(1-V_{f}\right)$$

where $E_{f}$ is elastic modulus of fiber; $E_{m}$ is elastic modulus of matrix; $V_{f}$ is volume fraction of fiber; $V_{m}$ is volume fraction of matrix.

When the fiber is laid along the reference direction (0${}^{\circ }$), according to the material performance parameters and volume fraction in Table 1, the longitudinal elastic modulus of the CFRP can be calculated as $E_{c1}=76,320$ MPa. The results are similar to a fiber unit cell, indicating that the elastic modulus of CFRP can be effectively calculated by the homogenization approach in ABAQUS. As shown in Equation (30), when the fiber is laid along 60${}^{\circ }$ and −60${}^{\circ }$, the elastic modulus of the fiber at fracture is shown as $E_{c2}=E_{f}V_{f}cos60^{\circ }+E_{m}\left(1-V_{f}\right)=40,520$ MPa. Take two layers (0${}^{\circ }$, 60${}^{\circ }$ or 0${}^{\circ }$, −60${}^{\circ }$) of fiber composite as 50% of the volume, then the overall elastic modulus is $E_{c}=\left(E_{c1}+E_{c2}\right)/2=58,420$ MPa.

##### Transverse Modulus

When the CFRP is subjected to transverse stress, the transverse displacement is equivalent to the sum of the transverse displacement of fiber and matrix,

(31) $$\delta_{c^{\prime }}=\delta_{f^{\prime }}+\delta_{m^{\prime }}$$

(32) $$\epsilon =\frac{\delta }{L}$$

(33) $$\sigma_{c^{\prime }}=\sigma_{f^{\prime }}=\sigma_{m^{\prime }}$$

then

(34) $$\delta_{c^{\prime }}=\epsilon_{c^{\prime }}L=V_{f}\epsilon_{f^{\prime }}L+V_{m}\epsilon_{m^{\prime }}L$$

invoking Hooke’s Law

(35) $$E_{c^{\prime }}=\frac{E_{f}E_{m}}{E_{f}V_{m}+E_{m}V_{f}}$$

where δ is displacement; *L* is original length.

The transverse elastic modulus of transverse fiber laying 90${}^{\circ }$ is shown as $E_{c3}=5835$ MPa. The elastic modulus of fibers laid along the direction of 60${}^{\circ }$ can be calculated after being subjected to transverse tension. The elastic modulus is $E_{c4}=E_{f}V_{f}sin60^{\circ }+E_{m}\left(1-V_{f}\right)=33,459$ MPa. Take the layers of 0${}^{\circ }$ direction and 60${}^{\circ }$ direction as superimposed together. Therefore, the elastic modulus of the laminated plate is shown as $E_{c^{\prime }}=\left(E_{c3}+E_{c4}\right)/2=19,647$ MPa.

In this study, the transverse elastic modulus of the fiber along the direction of 0${}^{\circ }$ and 60${}^{\circ }$ can be regarded as the longitudinal elastic modulus of the fiber along the direction of 90${}^{\circ }$ and 30${}^{\circ }$. The layout of the specimen used is [(0${}^{\circ }$/60${}^{\circ }$/0${}^{\circ }$/−60${}^{\circ }$)${}_{4}$], with 16 layers in total, which can be regarded as eight layers of 0${}^{\circ }$/60${}^{\circ }$. Then the elastic moduli of the total number of layers are the same as 0${}^{\circ }$/60${}^{\circ }$. Theoretically, the longitudinal and transverse elastic moduli are 58,420 MPa and 19,647 MPa respectively.

### 3.4. Experimental Results

#### 3.4.1. Longitudinal Uniaxial Stretching

**Figure 13:**
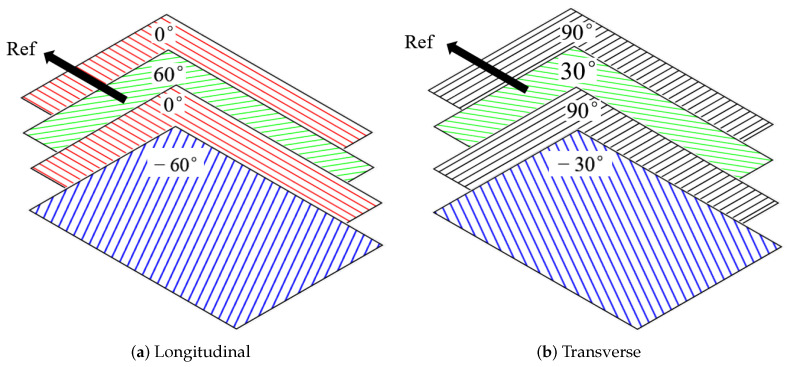
Two ways of uniaxial stretching.

In longitudinal uniaxial tension, the layout of the sample is [(0${}^{\circ }$/60${}^{\circ }$/0${}^{\circ }$/−60${}^{\circ }$)${}_{4}$], as shown in Figure 13a, and apply a load in the reference direction. In the experiment, the loading force is automatically recorded by the equipment, and the elongation of the specimen is recorded by the displacement meter.

**Figure 14:**
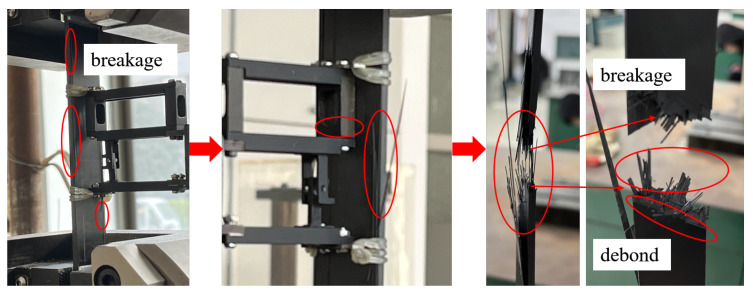
Longitudinal fracture specimen.

In the process of cutting the specimen, the inevitable damage causes the two sides of the specimen to break first. The fibers on both sides are the weakest, and a handful of epoxy is debonding at the same time. As the experiment goes on, the carbon fiber composite will fracture layer by layer. When all the fiber layers are broken, the specimen is completely destroyed. The results show that 0${}^{\circ }$ bedding is completely fractured, and the 60${}^{\circ }$ fiber layer is partially broken. Some of the interface is debonded along the fiber direction due to matrix cracking, as shown in Figure 14.

#### 3.4.2. Transverse Uniaxial Stretching

**Figure 15:**
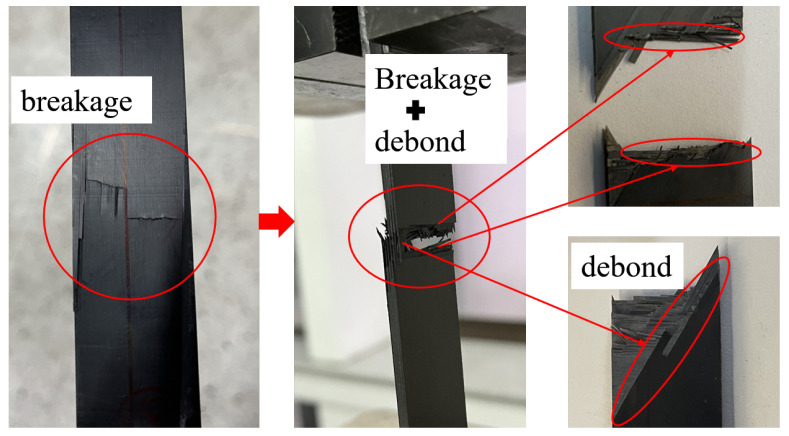
Transverse fracture specimen.

We changed the layout of specimens to measure the transverse elastic modulus of [(0${}^{\circ }$/60${}^{\circ }$/0${}^{\circ }$/−60${}^{\circ }$)${}_{4}$] layering, as shown in Figure 13b. The specimen is stretched along the reference direction, which is equivalent to the tensile test of the laminated plate [(90${}^{\circ }$/30${}^{\circ }$/90${}^{\circ }$/−30${}^{\circ }$)${}_{4}$]. The experiment is conducted in the same way as that of longitudinal stretching. The results are shown in Figure 15. Part of the fibers on both sides of the specimen is fractured first, which may be due to weak fibers on both sides caused by cutting or incomplete alignment of clamping, and then cracks and crack propagation occurred. The transverse propagation of the crack indicates that the interface between the fiber and the matrix at the direction of 90${}^{\circ }$ is degummed and then the composite layer is fractured. In the 90${}^{\circ }$ direction, the fracture is parallel to the fiber direction, and the force is borne by the epoxy. In the direction of 30${}^{\circ }$, part of the fibers have broken, and some fibers have interface degumming, so there are fibers laid parallel to 30${}^{\circ }$ at the fracture.

**Figure 16:**
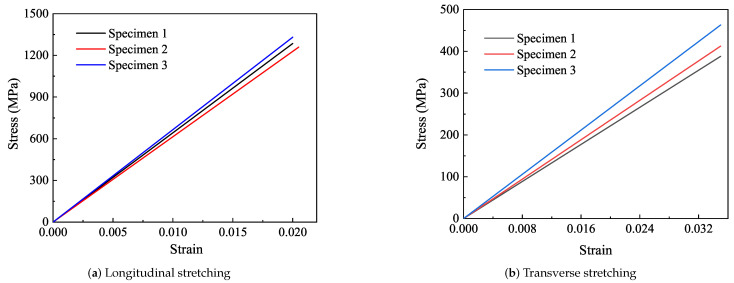
Stress–strain curve.

As a brittle material with excellent performance, CFRP is characterized by high strength and high modulus. The stress–strain curve of the longitudinal tensile sample is shown in Figure 16a. The maximum stress values of the three groups of effective specimens in the elastic range are 1285 MPa, 1259 MPa, and 1331 MPa, respectively, and the average stress is 1292 MPa. The result of the experiment is acceptable.

The transverse elastic modulus of the CFRP sample is obviously smaller than the longitudinal elastic modulus, which is due to the fact that the fiber bears less force and the epoxy bears more force in the transverse direction. The stress–strain curve of the transverse experiment is shown in Figure 16b. The maximum stress of three effective specimens in the elastic range are 388 MPa, 412 MPa, and 463 MPa, respectively, and the average stress is 412 MPa. By referring to Hooke’s Law, the elastic moduli of the three specimens are obtained respectively, and the average value is the transverse elastic modulus of the laminated plate, which is 12,033 MPa.

#### 3.4.3. Macroscopic Perspective

**Figure 17:**
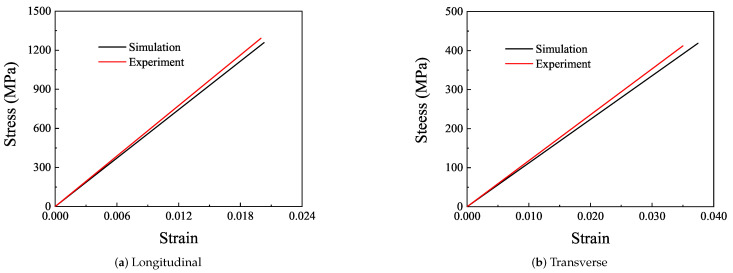
Macro simulation and test of stress and strain.

**Figure 18:**
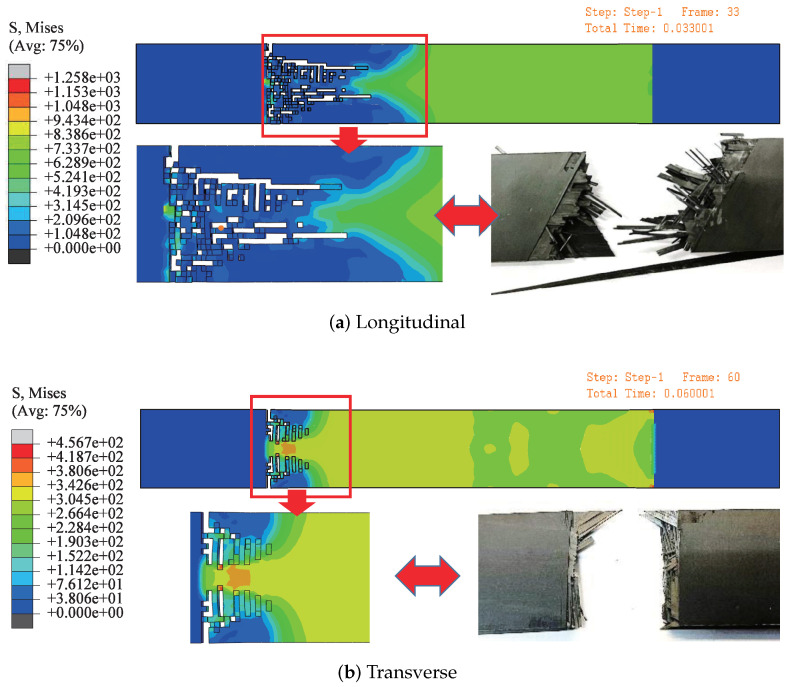
Comparison between simulation and experiment.

The homogenized material parameters are used in the simulation, and the results are shown in Figure 17. The longitudinal and transverse elastic moduli of the specimens are 64,583 MPa and 12,033 MPa, respectively. The longitudinal and transverse elastic moduli of the simulation model are 63,863 MPa and 11,317 MPa, respectively. The fracture simulation results of CFRP laminates are compared with the experiment, as shown in Figure 18.

The elastic modulus of the CFRP obtained from the experiment is larger than that obtained from the simulation. During the tensile process of specimens, the edges of the specimens are damaged firstly due to the alignment deviation and initial defect, which reduces the elongation of the specimen, and finally, a stronger elastic modulus is obtained in the experiment. The deviation of longitudinal elastic modulus between simulation and experiment is 1.11% and the transverse elastic modulus is 5.95%. The deviation of theory and test is 9.54 and 38.76%. There is a large deviation between theoretical and experimental transverse elastic modulus, which may be due to fiber debonding in the experiment, which is not considered in the theoretical calculation.

**Figure 19:**
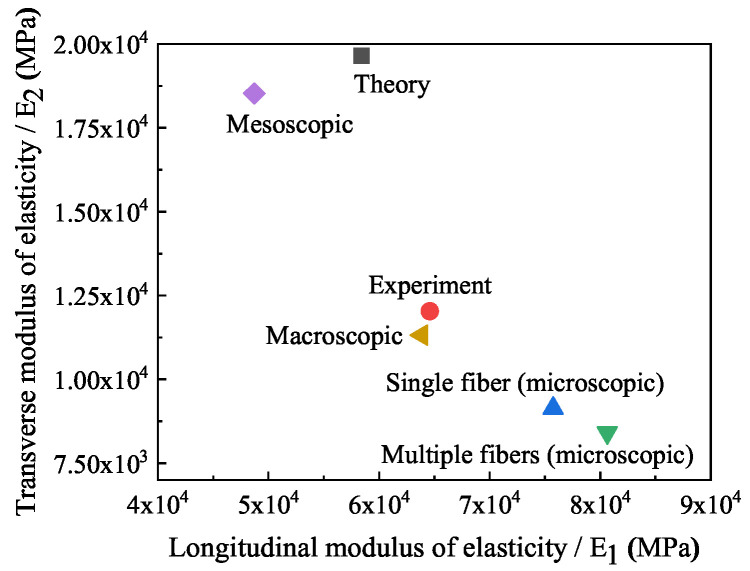
Elastic moduli at all scales, theoretical and experimental.

From the microscopic RVE of a fiber and multiple fibers, to the mesoscopic RVE, and finally, to the macroscopic, their elastic moduli are calculated respectively. Results of the bridging-scale analysis are shown in Figure 19. The theoretical values and mesoscopic simulation values are similar, but the experimental values are smaller because of the debonding of some fibers. For theory and experiment, in addition to one longitudinal elastic modulus and one transverse elastic modulus, the theoretical value of the elastic modulus in the third direction is 5834.6 MPa, and the fiber bearing force in the third direction is less.

The microscopic RVE has a larger elastic modulus when the load is applied along the fiber distribution direction, and the fibers endure greater forces than the matrix. In the mesoscopic model, the fiber distribution at 0${}^{\circ }$ and 60${}^{\circ }$ is adopted, as a whole. The elastic modulus of the layout is smaller than that of the composite layer laid at 0${}^{\circ }$ completely. After the material parameters calculated by the mesoscopic model are used in the macroscopic model, the error between the elastic modulus of the macroscopic model and the experimental results is reasonable, so the homogenized material parameters can be used in the macroscopic model to reduce the complexity of composite layer modeling.

## 4. Mechanical Analysis of Type iii Pressure Vessel

**Figure 20:**
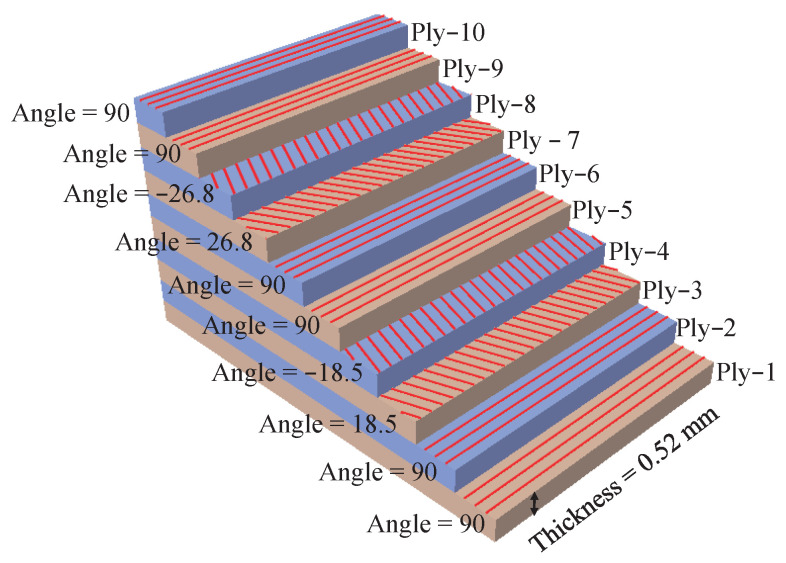
Fiber/epoxy composite layers.

In the study, the blasting pressure of composite pressure vessels is predicted based on the asymptotic homogenization approach from a macroscopic perspective. The failure mode is not considered. In order to verify validate simulation results, the model structure and material parameters in literature [39] are adopted. Type III hydrogen storage pressure vessels consist of aluminum liner and composite material layers. The lining and composite material parameters in literature [39] are shown in Table 4 and Table 5. The winding mode of composite layers is [90${}^{\circ}{\;}_{2}$/± 18.5${}^{\circ }$/90${}^{\circ}{\;}_{2}$/± 26.8${}^{\circ }$/90${}^{\circ}{\;}_{2}$]. The thickness of each layer of composite material is 0.52 mm, as shown in Figure 20.

**Figure 21:**
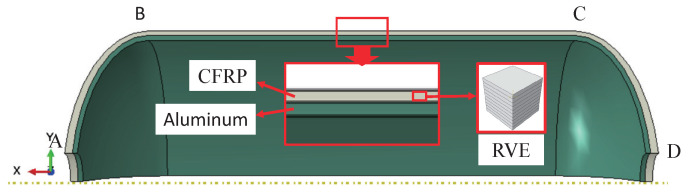
Simplified geometry model of type III pressure vessel, where A, B, C, and D refer to the vertices of the dome and cylinder.

The elastic moduli and Poisson’s ratio of RVE are calculated by RVE at the macro-scale using the homogenization approach. The homogenized material parameters are shown in Table 6. Using the same model parameters in literature [39], a pressure vessel model is established as shown in Figure 21. As the pressure vessel is an axisymmetric model, a quarter model of the pressure vessel is established for convenient calculation, with cyclic symmetric constraints and fixed constraints at both ends. The lining material of this model is aluminum, and the outside represents ten layers of composite material. The homogenized material parameters cannot show the damage to each layer, but the blasting pressure can be easily predicted. As a matter of experience, the first place to destroy the pressure vessel is the transition between the head and the cylinder, so the dome is simplified. The simplified treatment has the advantage of reducing the computational difficulty, at the same time requiring the prediction of burst pressure to be as unaffected as possible.

**Figure 22:**
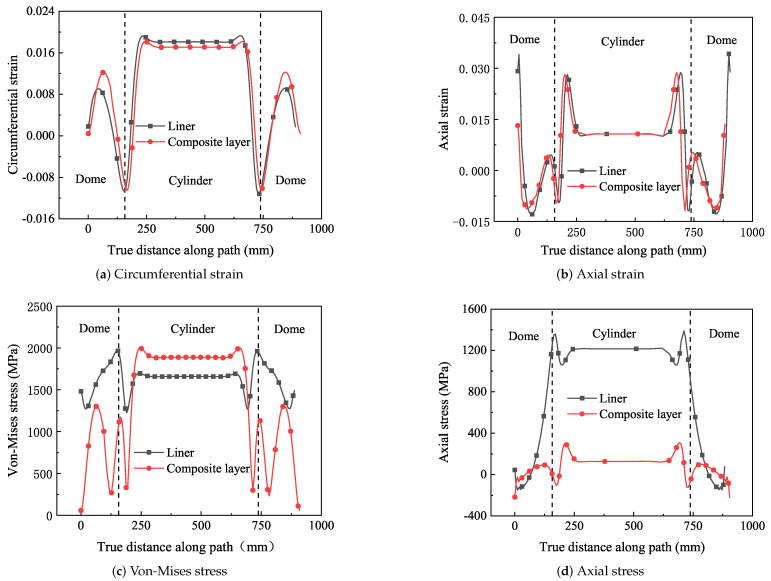
The stress and strain of composite vessel along the path A-B-C-D.

**Figure 23:**
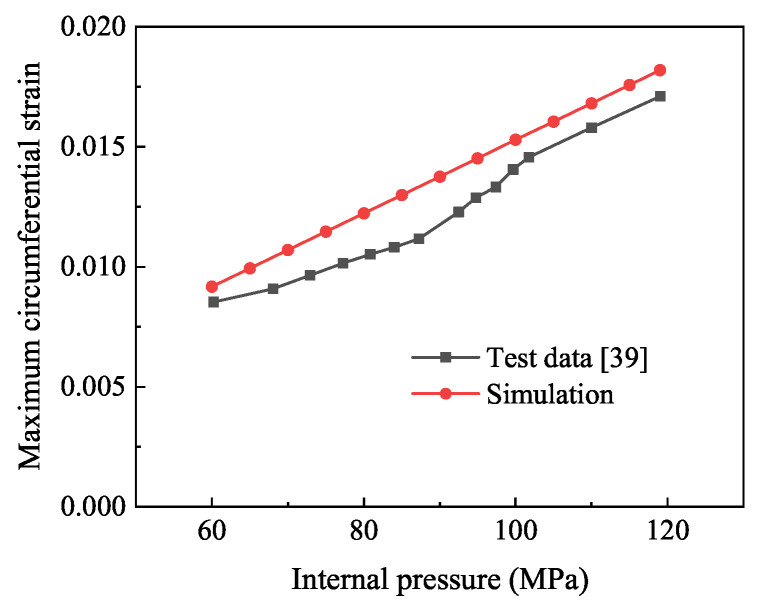
Comparison of circumferential strain between simulation and reference curve.

For the prediction of the bursting pressure of the composite pressure vessel, the best way is to apply the maximum strain criterion to the model with the increasing pressure of 0–119 MPa. Observe the numerical magnitude and distribution of circumferential strain and stress. On the path from one end head to the other end dome (A-B-C-D in Figure 21), the pressure inside the pressure vessel reaches 119 MPa, and the maximum circumferential strain of the composite layer occurs at the cylinder, and its value is 0.018. At this point, the strain value of the composite layer is about 85% of that of the independent fiber, as shown in Figure 22a, which is in good consistency with reference [39], as shown in Figure 23. The model is damaged at the shell and the transition firstly, and the circumferential strain of the liner at the dome is smaller than that composite layer, and the circumferential strain of the liner is slightly larger than that of the composite layer at the shell. Under normal circumstances, the blasting pressure is three-tenths of the standard pressure, so the standard pressure of the design should be below 35 MPa.

The stress distribution of the lining and composite layer is shown in Figure 22c. Due to homogenization, the microscopic stress distribution can not be seen in the results. The overall stress distribution can be calculated in ABAQUS. The stress of the composite layer is obviously greater than that of the lining on the cylinder. For the domes, simulation results may not be accurate due to simplification. It can be seen from Figure 22b,d that the axial strain of the lining and composite layer have the same change trend, and the axial stress value of the lining is much smaller than that of the composite layer.

**Figure 24:**
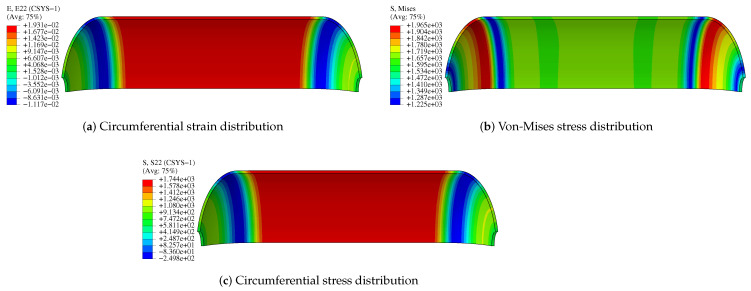
Stress and strain distribution of liner.

**Figure 25:**
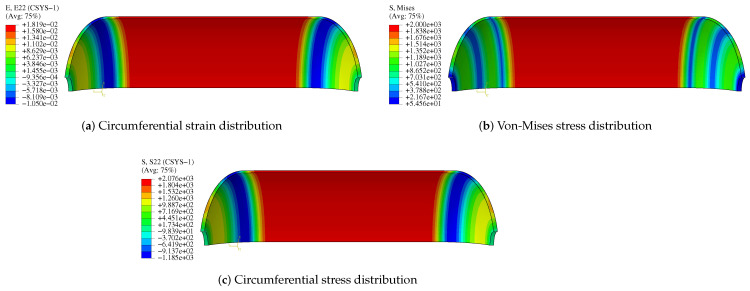
Stress and strain distribution of CFRP.

The results show that circumferential and axial displacements of composite pressure vessels occur under internal pressure. Circumferential strain and circumferential stress occur when the cylinder expands. The domes will produce stress and strain due to the action of internal pressure, but due to their special structure, the stress and strain of domes are smaller than the barrel segment, and the weakest region is located in the barrel segment and the transition. Figure 24 and Figure 25 show the stress and strain distribution of the liner and composite layer. The maximum von-Mises stress of the liner is located in the transition layer, but the maximum circumferential stress and strain of the liner are located in the cylinder. The maximum circumferential strain of the composite layer is located in the cylinder, and the strain of the transition layer is relatively large. Relevant experiments have proved that the damage to composite pressure vessels occurs in the cylinder [40], which is the same as the simulation results.

## 5. Conclusions

In this study, the thermal stress homogenization method-based asymptotic homogenization approach is used for multiscale analysis of CFRP. The homogenized material parameters of multilayer CFRP structures are calculated by using ABAQUS and subroutines. From the macroscopic perspective, the tensile experiments of [${\left(0^{\circ }/60^{\circ }/0^{\circ }/-60^{\circ }\right)}_{4}$] CFRP structure are carried out to validate the homogenized material parameters in predicting the ultimate strength. The CFRP laminates of asymptotic homogenization are applied to model the filament winding of type III pressure vessel. Findings are drawn as follows:

(1) From a micro perspective, the homogenized elastic moduli of CFRP with the same fiber volume fraction but different fiber numbers have some deviation but are within an acceptable range. The homogenized elastic moduli and homogenized Poisson’s ratio are reasonable. At the mesoscopic level, it is not necessary to establish all the layering models but to establish partial structural models that can reflect the layering laws, so as to predict the overall structural parameters.
(2) The simulation results from a macroscopic perspective are consistent with the uniaxial tensile test results of the specimens, indicating that the ultimate strength and elastic moduli of CFRP structures can be predicted without considering the failure modes inside the structure.
(3) A method to solve the properties of CFRP structures is developed by combining the processive homogenization approach with ABAQUS finite element analysis. Applying this method to type III pressure vessels, the complexity of the model can be simplified, and the prediction result of burst pressure is reasonable. The cylinder and the transition region of the pressure vessel will be destroyed first, and the distributions of stress and strain can also be predicted.

## Figures and Tables

**Table 1:** Material property.

	Density $\left(g/{\mathrm{cm}}^{3}\right)$	E (MPa)	ν
T700SC-12K	1.8	230,000	0.3
914 epoxy	1.69	4000	0.39

**Table 2:** Material properties of RVE in homogenization.

	$E_{1}\left(\mathrm{MPa}\right)$	$E_{2}\left(\mathrm{MPa}\right)$	$E_{3}\left(\mathrm{MPa}\right)$	$\nu_{12}$	$\nu_{13}$	$\nu_{23}$
A fiber	75,757.58	9132.42	9132.42	0.36	0.36	0.51
Fibers	80,645.16	8410.43	8703.22	0.37	0.37	0.54

**Table 3:** Homogenized engineering constants of mesoscopic carbon fiber composites.

$E_{1}\left(\mathrm{MPa}\right)$	$E_{2}\left(\mathrm{MPa}\right)$	$E_{3}\left(\mathrm{MPa}\right)$	$\nu_{12}$	$\nu_{13}$	$\nu_{23}$	$G_{12}\left(\mathrm{MPa}\right)$	$G_{13}\left(\mathrm{MPa}\right)$	$G_{23}\left(\mathrm{MPa}\right)$
48,702.089	10,630.608	18,525.380	0.36	0.29	0.23	3229.035	4418.718	3624.764

**Table 4:** Mechanical properties parameters of T700/epoxy composite layer.

T700/epoxy	$E_{L}\left(\mathrm{GPa}\right)$	$E_{T}\left(\mathrm{GPa}\right)$	$\nu_{\mathrm{LT}}$	$\nu_{\mathrm{TT}}$	$G_{\mathrm{LT}}\left(\mathrm{GPa}\right)$	$X_{T}\left(\mathrm{MPa}\right)$	$X_{C}\left(\mathrm{MPa}\right)$	$Y_{T}\left(\mathrm{MPa}\right)$	$Y_{C}\left(\mathrm{MPa}\right)$	S(MPa)
Value	181	10.3	0.28	0.49	5.17	2150	2150	298	298	778

**Table 5:** Mechanical property of 6061-Al.

Aluminum(6061)	E(GPa)	ν	$\sigma_{s}\left(\mathrm{MPa}\right)$	$\sigma_{b}\left(\mathrm{MPa}\right)$
Value	70	0.3	246	324

**Table 6:** Material parameters of homogenized CFRP.

$E_{1}\left(\mathrm{MPa}\right)$	$E_{2}\left(\mathrm{MPa}\right)$	$E_{3}\left(\mathrm{MPa}\right)$	$\nu_{12}$	$\nu_{13}$	$\nu_{23}$	$G_{12}\left(\mathrm{MPa}\right)$	$G_{13}\left(\mathrm{MPa}\right)$	$G_{23}\left(\mathrm{MPa}\right)$
112,359.551	10,288.066	8928.571	0.17	0.18	0.46	5173.306	1484.781	5540.166

## Data Availability

Not applicable.

## Sample Availability

Samples of the compounds are available from Qi Wu of the Nanjing University of Aeronautics and Astronautics.
